# A strategy for L-isoleucine dioxygenase screening and 4-hydroxyisoleucine production by resting cells

**DOI:** 10.1080/21655979.2017.1304872

**Published:** 2017-04-28

**Authors:** Chenglin Zhang, Jie Ma, Zhixiang Li, Yunlong Liang, Qingyang Xu, Xixian Xie, Ning Chen

**Affiliations:** a College of Biotechnology, Tianjin Engineering Lab of Efficient and Green Amino Acid Manufacture, National and Local United Engineering Lab of Metabolic Control Fermentation Technology, Tianjin University of Science and Technology, Tianjin, China; b Linghua Group Limited, Shandong, China

**Keywords:** 4-hydroxyisoleucine, L-isoleucine dioxygenase, resting cells, succinate

## Abstract

L-Isoleucine dioxygenase (IDO) specifically converts L-isoleucine(L-Ile) to 4-hydroxyisoleucine(4-HIL). To obtain IDO with improved activity, a strategy was developed that is dependent on the restoration of succinate-minus *E. coli* cell growth by the coupling of L-Ile hydroxylation and the oxidation of α-ketoglutarate(α-KGA) to succinate. Five mutants were obtained with this strategy, and the characteristics of IDO^M3^, which exhibited the highest activity, were studied. The catalytic efficiency, thermal stability and catalytic rate of IDO^M3^ were significantly improved compared with those of wild-type IDO. Moreover, an efficient method for the biotransformation of 4-HIL by resting cells expressing IDO^M3^ was developed, with which 151.9 mmol of 4-HIL/L (22.4 g/L) was synthesized in 12 h while the substrates seldom exhibited additional consumption.

## Introduction

4-Hydroxyisoleucine(4-HIL) is a natural nonproteinogenic amino acid that was first isolated from the seeds of *Trigonella foenum-graecum* and has been proven to exhibit glucose-dependent insulinotropic activity in rat models of type 2 diabetes mellitus(T2DM). Moreover, 4-HIL does not induce side effects such as hypoglycemia, which occurs during T2DM therapy.^1-4^ 4-HIL has also been found to be an effective drug to control body weight gain, glycemia, insulinemia and decreased plasma triglyceride levels in rodents.^5^ Furthermore, recent studies have shown that 4-HIL exhibits effective antidiabetic activity in a model of type1 diabetes mellitus without insulin.^6^

The primary method for 4-HIL production is extraction from fenugreek seeds; however, the yield by this method is rather low (approximately only150 mg of 4-HIL can be extracted from 1 kg of fenugreek seeds).^2,7^ In addition to seed extraction, chemical and enzymatic synthesis methods have also been developed but were considered to result in lowefficiency, highcost and heavy pollution^8-10^

4-HIL has at least 8 stereo configurations, but only (2S, 3R, 4S)-4-HIL exhibits biologic activity.^11^ L-Isoleucine dioxygenase (IDO) from *Bacillus thuringiensis* 2e2 has been found to specifically convert L-isoleucine(L-Ile) to (2S, 3R, 4S)-4-HIL, which is a member of the α-ketoglutarate(α-KGA)-dependent hydroxylase family, and (2S, 3R, 4S)-4-HIL has been successfully synthesized by IDO.^11^ In a previous study, we cloned *ido*(Accession KC884243) from isolated *B. thuringiensis* TCCC 11826, and IDO was used to synthesize 4-HIL via biotransformation.^12^ However, the 4-HIL production was rather low(44.64 mM in 36 h), and the substrates(L-Ile and α-KGA) were also found to exhibit additional consumption during the process of biotransformation.

To further gain IDO with improved activity, a strategy dependent on the coupling of L-Ile hydroxylation, the oxidation (decarboxylation) of α-KGA to succinate and cell growth was developed. Five mutants were obtained via this strategy, and the characteristics of the mutant exhibiting the highest activity were studied. Moreover, a method for 4-HIL production by resting *Escherichia coli* cells overexpressing activity-improved IDO was developed.

## Results and discussion

### Restoration of *E. coli* ΔsucAΔaceA-ido growth by IDO via the coupling of L-Ile hydroxylation and the oxidation of α-KGA to succinate

One unique property of the α-KGA-dependent hydroxylase reaction is the coupling of substrate hydroxylation and the oxidation (decarboxylation) of α-KGA to succinate.^13^ Thus, IDO may shunt the TCA cycle when succinate synthesis is blocked, thereby coupling L-Ile hydroxylation and cell growth.

**Figure 1.**
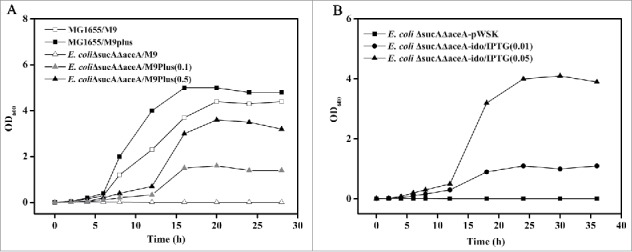
Growth of *E. coli* cells under different conditions. (A) *E. coli* K-12 MG1655 grown on M9 medium(open squares) or M9 medium supplemented with succinate(solid squares); *E. coli* ΔsucAΔaceA grown on M9 medium(open triangles), M9 medium supplemented with 0.1 g succinate/L (gray solid triangles) or M9 medium supplemented with 0.5 g succinate/L (black solid triangles). (B) *E. coli* ΔsucAΔaceA harboring pWSK29(*E. coli* ΔsucAΔaceA-pWSK) grown on M9 medium supplemented with 1 g/L L-Ile and α-KGA(solid squares) or *E. coli* ΔsucAΔaceA harboring pWSK-ido(*E. coli* ΔsucAΔaceA-ido) grown on the same medium but under the induction of 0.01 mM(solid circles) or 0.05 mM(solid triangles) IPTG.

In *E. coli*, there are 4 pathways for succinate synthesis: (1) from α-KGA by α-ketoglutarate dehydrogenase via the TCA cycle under aerobic conditions, (2) from isocitrate by isocitrate lyase via the glyoxylate pathway, (3) from oxaloacetate through the reductive branch of the TCA cycle under anaerobic conditions, and (4) from L-glutamate and L-arginine via the γ-aminobutyrate catabolic pathway.^14^ To block succinate synthesis in *E. coli* K-12 MG1655, both the *sucA*-encoding submit of α-ketoglutarate dehydrogenase and *aceA*-encoding isocitrate lyase were deleted, resulting in *E. coli* ΔsucAΔaceA. The plasmid pWSK-ido was subsequently introduced into *E. coli* ΔsucAΔaceA, and *E.coli*ΔsucAΔaceA-ido was obtained. *E. coli* ΔsucAΔaceA could not grow in M9 medium without the addition of succinate during aerobic cultivation although the γ-aminobutyrate catabolic pathway was still retained (Fig. 1A). The effect of succinate addition on the growth of *E.coli*ΔsucAΔaceA was detected, and the biomass of *E.coli*ΔsucAΔaceA was enhanced with the increased addition of succinate. Notably, *E.coli*ΔsucAΔaceA-ido induced by IPTG restored growth in M9 medium supplemented with L-Ile and α-KGA, indicating that IDO activity shunts the blocked TCA cycle and restores cell growth coupling with L-Ile hydroxylation (Fig. 1B).

4-HIL accumulation was detected in culture broth during cell growth. The biomass (Fig. 1B) and 4-HIL accumulation(data not shown) were enhanced with the increase in IPTG addition. Because 4-HIL accumulation is coupled with succinate synthesis, it was deduced that the higher biomass was due to increased succinate synthesis catalyzed by the enhanced expression of *ido* induced by an increased IPTG concentration.

### Screening the IDO variants with improved activities

**Figure 2.**
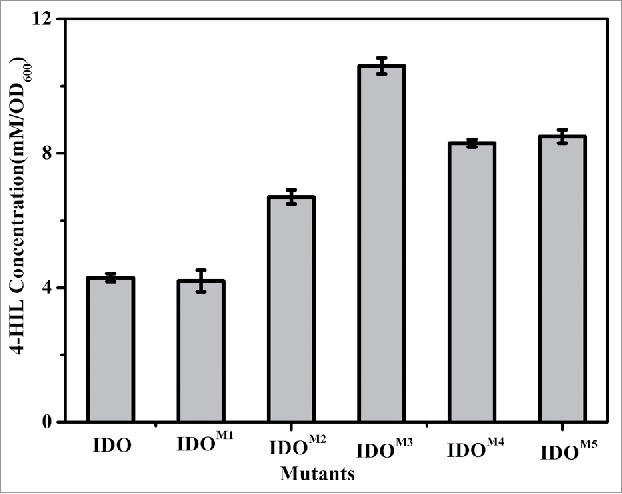
Production of 4-HIL by *E. coli* ΔsucAΔaceA harboring *ido* variants in 24-well microplates. The error bars represent the SD of the mean calculated for 3 replicates.

Because IDO can couple L-Ile hydroxylation and *E.coli*ΔsucAΔaceA cell growth and because the biomass was dependent on the level of succinate synthesis, one would expect that a higher IDO activity would correspond to a greater supply of succinate and larger colonies of strains expressing *ido* on the plates. Error-prone PCR was performed with *ido* as a template, and the mutated gene products were subsequently ligated into the vector pWSK29. The recombinant plasmids were transformed into *E.coli*ΔsucAΔaceA to obtain a mutant library. After the screening of approximately 9,000 clones, 5 were found to show considerably larger colonies than the wild type. The 5 clones were collected, and 4 clones repeatedly showed increased 4-HIL production compared with the strains with wild-type IDO, indicating improved IDO activity (Fig. 2).

Sequence analysis of IDO^M3^, which exhibited the highest IDO activity, showed that the residues Leu27, Glu80, Gly169 and Ser182 were substituted for Ile27, Asp80, His169 and Asp182, respectively. The active site structures of α-KGA-dependent hydroxylase exhibit nearly identical arrangements of the 3 amino acid side chains, a His^1^-X-Asp/Glu-Xn-His^2^ motif.^13^ As a member of the superfamily, IDO also has the only His^159^-X-Asp^161^-X_50_-His^212^ motif, which might be the active site. Thus, one could reason that the 2 mutated amino acid residues(Gly169 and Ser182) located in the motif resulted in the improved activity of IDO^M3^.

### Expression and characterization of IDO^M3^

*Ido*^M3^ was expressed by *E. coli* BL-ido^M3^ under the induction of IPTG, and recombinant IDO ^M3^ was purified by a Ni^2+^-NTA affinity column. The concentrations of the purified recombinant IDO and IDO^M3^ were 5.1 and 4.2 mg/mL, respectively. To calculate the K_m_, k_cat_ and V_max_ values of IDO and IDO^M3^, the activities of the 2 enzymes toward different concentrations of L-Ile were measured, and the data were plotted according to the Michaelis-Menten equation. As shown in Table 1, K_m_ was lower, but V_max_ and K_cat_ were higher for IDO^M3^ than IDO. The catalytic efficiency k_cat_/K_m_ was approximately 1.5-fold higher for IDO^M3^ than for IDO under the measured conditions.

**Table 1.** Kinetic apparent constants determined for IDO and IDO^M3^.

Enzymes	K_m_(mM)	K_cat_(1/min)	K_cat_/K_m_(L/mmol/min)	V_max_(umol/min/mg)
IDO	0.20	4.36	21.80	2.18
IDO^M3^	0.15	5.02	33.47	2.51

**Figure 3.**
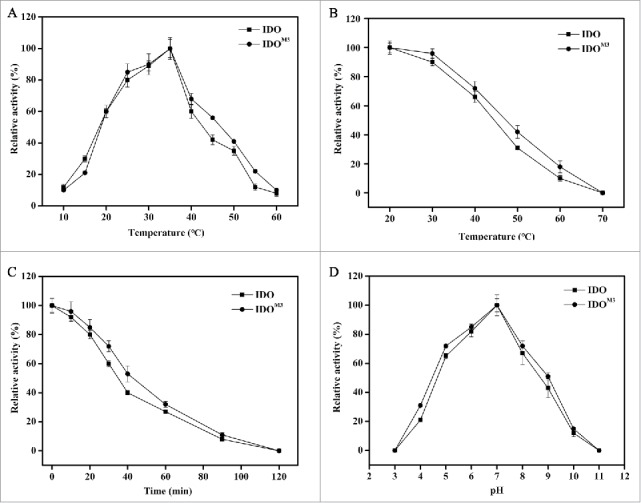
Effect of temperature and pH on enzyme activity and stability. (A) Effect of temperature on enzyme activity. The maximum activity at 35°C was taken as 100%. (B) Effect of temperature on enzyme stability. Enzymes were incubated for 1 h at the indicated temperatures. The samples were then measured under standard conditions. The activity without treatment was taken as 100%. (C) Time-course thermal stability. The enzyme activities were determined by evaluating residual activities after incubation for 0 min, 10 min, 20 min, 30 min, 40 min, 60 min, 90 min and 120 min at 60°C. The activity without treatment was taken as 100%. (D) Effect of pH on enzyme activity. The enzyme activity was measured at 35°C in different buffers with pH values ranging from 3 to 11. The maximum activity observed in pH 7.0 buffer was taken as 100%. The error bars represent the SD of the mean calculated for 3 replicates. The circles represent IDO, and the squares represent IDO^M3^.

The optimum temperature for the activity of IDO^M3^ and IDO was determined within a range of 10°C to 60°C. As shown in Fig. 3A, the optimum temperature for both enzymes was approximately 35°C. Thermal stability assays showed that both IDO and IDO^M3^ were stable below 40°C, while the activities decreased dramatically when the temperature was above 40°C (Fig. 3B). More than 60% of the maximum activity was observed after incubation at 60°C for 30 min (Fig. 3C). Notably, IDO^M3^ exhibited a slightly higher stability than IDO. After incubation at 60°C for 40 min, 53% of IDO^M3^ activity was retained, compared with 40% of IDO activity (Fig. 3C). The mutated amino acid residues at sites 27 and 80 may be responsible for the improved thermal stability. The optimum pH for the activity of IDO^M3^ and IDO was determined over a pH range of 3.0 to 11.0. The activity of the 2 enzymes rose with the increased pH until 7.0 and then decreased, with no difference in the optimum pH for the 2 enzymes (Fig. 3D).

### 4-HIL synthesis by resting cells expressing IDO^M3^

**Figure 4.**
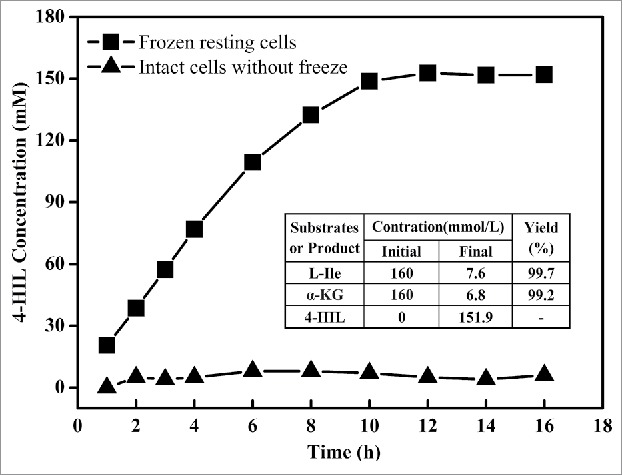
Production of 4-HIL by *E. coli* ΔsucAΔaceA-ido^M^ cells subjected to freezing (squares) or not subjected to freezing (triangles). The final concentrations of L-Ile, α-KGA and 4-HIL as well as yield in the table were detected at 12 h of the process, when the 4-HIL production reached its peak value.

Under industrial application conditions, purified enzymes are not a suitable enzyme source. Biotransformation by *E. coli* harboring *ido* seems to be an appropriate method for 4-HIL synthesis. However, 50 nmol of glucose (150 nmol in total) was additionally consumed for cell growth rather than for 4-HIL synthesis during process biotransformation.^15^ Moreover, additional consumption of L-Ile and α-KGA was also detected in our previous study(data not shown). One attractive solution would be to use resting cells, a strategy that has been successfully used in nitrile hydrolase biocatalysis.^16^ However, the cell membrane seems to be the main limitation for the uptake of L-Ile and α-KGA and the export of 4-HIL. Thus, to improve the cell permeability of the substrate and products, harvested *E. coli* BL21(DE3) cells expressing IDO^M3^ were frozen at −80°C to obtain resting cells, and the strategy for 4-HIL synthesis was evaluated. As shown in Fig. 4, the resting cells could successfully transform L-Ile to 4-HIL, while intact cells that had not been frozen were not successful. Next, 151.9 mmol of 4-HIL/L (22.4 g/L) was synthesized in 12 h, with a yield of 0.997 mmol 4-HIL/mmol L-Ile and 0.992 mmol 4-HIL/mmol α-KGA, concentrations that are significantly higher than those obtained by the biotransformation method used previously.^12^ According to the reaction stoichiometry, this process requires 151.9 mM L-Ile and α-KGA; the actual values were 152.4 mM and 153.2 mM, respectively, similar to the theoretical values. The results indicate that L-Ile and α-KGA were seldom subject to additional consumption during the process of biotransformation. Furthermore, the resting cells could be collected after the biotransformation and recycled at least 4 times while exhibiting almost the same yield (data not shown).

## Materials and methods

### Strains and plasmids

The strains and plasmids used in this work are listed in Table 2.

**Table 2.** Strains and plasmids.

Strains	Relevant characteristics	Source
*E. coli* BL21(DE3)	Wild type	Laboratory stock
*E. coli* K-12 MG1655	Wild type	Laboratory stock
*E. coli* ΔsucA	*E. coli* K-12 MG1655 with deletion of *sucA*	This study
*E. coli* ΔsucAΔaceA	*E. coli* K-12 MG1655 with deletion of *sucA* and *aceA*	This study
*E. coli* ΔsucAΔaceA-pWSK	*E. coli* ΔsucAΔaceA harboring pWSK29	This study
*E. coli* ΔsucAΔaceA-ido	*E. coli* ΔsucAΔaceA harboring pWSK-ido	This study
*E. coli* ΔsucAΔaceA-ido^M^	*E. coli* ΔsucAΔaceA harboring pWSK-ido ^M3^	This study
BL-ido	*E. coli* BL21(DE3) harboring pET-ido	This study
BL- ido^M3^	*E. coli* BL21(DE3) harboring pET-ido ^M3^	This study
Plasmids		
pET-His	Expression vector, Amp^r^	Laboratory stock
pWSK29	Expression vector, Amp^r^	Laboratory stock
pKD3	As a template for amplification of chloramphenicol resistance gene cassette, Amp^r^, Chl^r^	Laboratory stock
pWSK-ido	pWSK29 containing *ido*	This study
pWSK-ido ^M3^	pWSK29 containing *ido*^M3^	This study
pET-ido	pET-His containing *ido*	Laboratory stock
pET-ido^M3^	pET-His containing *ido*^M3^	This study

### Primers

The primers used in this work are listed in Table 3.

**Table 3.** Primers.

Primers^*^	Sequence (5′–3′)	Description
sucA-1	AAGCATAAAAAAGATGCTTAAGGGATCACGTAACGGCTGACATGGGAATTAG^*^	Deletion of *sucA*
sucA-2	TATCTACGCTACTCATTGTGTATCCTTTATGCAACAACAACCGTTGCTGACTGTAGGCCG	
sucA-3	ACGTAGACAAGAGCTCGCAAGTG	Identification for *sucA* deletion
sucA-4	CGGGTTTTTTATGCCAGGTTG	
aceA-1	AACCACCACATAACTATGGAGCATCTGCACTAACGGCTGACATGGGAATTAG	Deletion of *aceA*
aceA-2	CGGCCTACAGTCAGCAACGGTTGTTGTTGCGCAACAACAACCGTTGCTGACTGTAGGCCG	
aceA-3	TGGAACAGATCACCACTTCCG	Identification for *aceA* deletion
aceA-4	ATGATAAGACGCGCAAGCGT	
ido-1	AAGGAAGCTAGATATGAAAATGAGTGGCTTTAGCATAG	Amplification of *ido* and expressed by pWSK29
ido-2	TTATTTTGTCTCCTTATAAGAAAATGTTACTA	
ido-3	ATGAAAATGAGTGGCTTTAGCATAG	Amplification of *ido* and expressed by pET-His
ido-4	TTATTTTGTCTCCTTATAAGAAAATGTTACTA	

* Underlined letters indicate the sequence 100 bpupstream or downstream of the gene to be deleted.

### Construction of *E. coli* ΔsucAΔaceA

Knockout of *sucA* and *aceA* was performed by PCR-based λ-red recombination as described previously.^17^ A 1114-bp DNA fragment containing the chloramphenicol resistance gene cassette from pKD3 was amplified using primers sucA-1 and sucA-2.The PCR products were electroporated into *E. coli* K-12 MG1655 carrying the λ-red recombinase expression plasmid pKD46. Cells in which homologous recombination occurred were selected on an agar plate containing chloramphenicol and were identified by direct colony PCR using primers sucA-3 and sucA-4. The antibiotic marker was eliminated using the helper plasmid pCP20. The resulting strain was denoted *E. coli* ΔsucA. *aceA* in *E. coli* ΔsucA was deleted with the same method, resulting in *E. coli* ΔsucAΔaceA.

### Construction of an *ido* mutant library and *ido* overexpression strain

A mutant library of the *ido* gene was constructed by error-prone PCR from pET-ido using an instant error-prone PCR kit(Tiandz, Inc.; 101005) and the primers ido-1 and ido-2. The mutated gene products were consequently ligated into the vector pWSK29, which had been digested by *Xba* I via the ClonExpressTM II One Step Cloning Kit(Vazyme Biotech Co., Ltd; C11201), using a homologous recombinase. The recombinant plasmids were transformed into *E. coli* ΔsucAΔaceA, and the cells were grown on plates containing M9 medium(glycerol as a carbon source) supplemented with L-Ile and α-KGA(1 g/L) as well as ampicillin(100 mg/L) and IPTG(0.05 mM).

The *ido* gene was amplified from pWSK-ido or pWSK-ido ^M3^ by PCR using primers ido-3 and ido-4. The obtained PCR products were digested by *Bam*H I and *Hind* III and were cloned into the expression vector pET-His. The recombinant plasmids(pET-IDO and pET-IDO^M3^) were further transformed into competent *E. coli* BL21(DE3) cells, and the cells were selected on LB plates containing 100 mg/L ampicillin, resulting in BL-ido and BL-ido^M3^.

### Screening IDO mutants with improved activity

The clones exhibiting larger colonies than the wild type were transferred to 24-well microplates with 800 μL of M9 medium supplemented with L-Ile(1 g/L), α-KGA(1 g/L) and IPTG(0.05 mM). After incubation at 37°C for 24 h, 40 μL of the culture in each well was then inoculated into the corresponding well of another 24-well microplate with the same medium and was then cultured for another 24 h. The biomass(OD_600_)was detected, and variants exhibiting a higher biomass were chosen for further analysis.

### Biotransformation of 4-HIL by *E. coli* ΔsucAΔaceA harboring *ido* variants and resting cells of BL- ido^M3^

*E. coli* ΔsucAΔaceA cells harboring *ido* variants were cultured in 24-well microplates with M9 medium supplemented with L-Ile(1 g/L), α-KGA(1 g/L) and IPTG(0.05 mM) at 37°C for 24 h, and then 0.1 mL of the culture broth was transferred to another 24-well microplate with the same medium, was cultured for another 24 h and was centrifuged at 4°C for 5 min. Five hundred microliters of supernatant was collected for a quantitative analysis of 4-HIL.

BL- ido^M3^ cells were harvested after induction by IPTG for 6 h and were frozen at −80°C for 2 h to obtainresting cells. The cells (20 g) were suspended in a 1-L shake flask with 200 mL of Tris-HCl (100 mM, pH 7.0) containing 160 mM L-Ile and α-KGA, 5 mM FeSO_4_ and 10 mM ascorbic acid; the cells were thenshaken at 37°C at 200 rpm for 16 h.

### Protein expression and purification

IPTG (0.1 mM) was added when the BL-ido and BL-ido^M3^ cells were grown in LB medium(with 100 mg/L ampicillin) to the midexponential stage. The cells were harvested by centrifuging and broken with sonication after cultivation for another 4 h. The expression of recombinant IDOs was confirmed by sodium dodecyl sulfate-polyacrylamide gel electrophoresis (SDS-PAGE). The 6×His-tagged IDOs were purified using a Ni^2+^-NTA affinity column. The concentration of the purified proteins was determined by the Bradford method using bovine serum albumin as a standard.

### Characterization of IDO and IDO^M3^

For a catalytic characterization of IDO and IDO^M3^, the reaction mixture composed of 10 mM α-KGA and L-Ile, 5 mM FeSO_4_, 10 mM ascorbic acid and 100 mM Tris-HCl(pH 7.0) was combined with 0.5 mg/mL of purified recombinant IDO, and the reaction was performed at 30°C for 30 min. The enzymatic activity was determined by the production of 4-HIL, as measured by high-performance liquid chromatography(HPLC).

The optimal temperatures for IDO and IDO^M3^ were determined by evaluating the activities at temperatures ranging from 10°C to 70°C. The thermal stability of the enzymes was determined by assessing their residual activities after incubation at temperatures from 10°C to 60°C for 1 h. The time-course thermal stability of the enzymes at 60°C was determined by evaluating their residual activities after incubation for 0 min, 10 min, 20 min, 30 min, 40 min, 60 min, 90 min and 120 min.

To determine the K_m_ values, L-Ile was used at concentrations of 0.02 to 5 mM. To examine the pH dependency of the reaction, citric acid sodium-citrate buffer (pH 3.0–5.0), phosphate-buffered saline (pH 6.0–8.0), Tris-HCl (pH 9.0) and sodium bicarbonate-sodium carbonate buffer (pH 10.0–11.0) were used.

### Analytical determination of 4-HIL concentration by HPLC

Four-HIL was analyzed by precolumn derivatization using 2, 4-fluoro-dinitrobenzene and was detected by HPLC using an Agilent C18 column (150 mm × 4.6 mm, 3.5 μm). Elution was performed using a gradient of 50% acetonitrile (v/v)/50mM (CH_3_COONa), fed at a constant flow rate of 1.0 mL/min. UV absorption was measured at 360 nm, and the column temperature was maintained at 33°C.

## Conclusions

A strategy for the selection of IDO with improved activity was developed based on the coupling of L-Ile hydroxylation, the oxidation of α-KGA to succinate and cell growth. Five mutants were obtained using this strategy. The catalytic efficiency, thermal stability, and catalytic rate of the IDO^M3^ variant selected by the strategy were significantly improved compared with those of wild-type IDO. A method for 4-HIL biotransformation by resting cells expressing IDO^M3^ was developedand was shown to be suitable for 4-HIL synthesis with substrates only seldom being subjected to additional consumption.
